# Association between static stretching load and changes in the flexibility of the hamstrings

**DOI:** 10.1038/s41598-021-01274-7

**Published:** 2021-11-05

**Authors:** Kosuke Takeuchi, Kazunori Akizuki, Masatoshi Nakamura

**Affiliations:** 1 Department of Physical Therapy, Kobe International University, Kobe, Hyogo Japan; 2 Institute for Human Movement and Medical Sciences, Niigata University of Health and Welfare, Niigata, Niigata Japan

**Keywords:** Preventive medicine, Rehabilitation

## Abstract

The purpose of the present study was to examine the association between static stretching load and changes in the flexibility of the hamstrings. Twelve healthy men received static stretching for 60 s at two different intensities based on the point of discomfort (100%POD and 120%POD intensity), in random order. To assess the flexibility of the hamstrings, the knee extension range of motion (ROM). Passive torque at end ROM, and muscle–tendon unit stiffness were measured before and after stretching. The static stretching load was calculated from the passive torque throughout static stretching. The knee extension ROM and passive torque at end ROM increased in both intensities (*p* < 0.01). The muscle–tendon unit stiffness decreased only in the 120%POD (*p* < 0.01). There were significant correlations between the static stretching load and the relative changes in the knee extension ROM (r = 0.56, *p* < 0.01) and muscle–tendon unit stiffness (r = − 0.76, *p* < 0.01). The results suggested that the static stretching load had significant effects on changes in the knee extension ROM and muscle–tendon unit stiffness of the hamstrings, and high-intensity static stretching was useful for improving the flexibility of the hamstrings because of its high static stretching load.

## Introduction

Static stretching is recommended to be included as part of a fundamental warm-up program to prevent muscle–tendon injuries^1^. In order to prevent muscle–tendon injuries such as muscle strain, it is important to decrease muscle–tendon unit stiffness^2–4^. When static stretching is performed at an intensity of the point of discomfort (POD), which is normal stretching intensity, it has been shown that 180 and 120 s of static stretching are required to decrease the muscle–tendon unit stiffness in the hamstrings^5,6^ and muscle stiffness in the medial gastrocnemius^7^, respectively, which are the common sites for muscle strain^8–11^. However, previous studies reported that conditioning coaches use static stretching for approximately 20 s as a part of their warm-up program^12,13^. It is very difficult to use static stretching for more than 120 s in a warm-up program, because the time of sports practice is very limited for many athletes. Therefore, it is necessary to develop a method of static stretching that can decrease the muscle–tendon unit stiffness in a shorter duration.

Recently, it is reported that high-intensity static stretching effectively decreases the muscle–tendon unit stiffness of the hamstrings in a short duration^14–16^. For example, Kataura et al.^14^ compared the effects of 180 s of three different intensities of static stretching (80, 100, 120%ROM) and showed that high-intensity static stretching (120%ROM) was most effective for decreasing the muscle–tendon unit stiffness of the hamstrings. Moreover, Takeuchi and Nakamura^15,16^ reported that high-intensity static stretching at the intensity of 120%POD for 20 or less seconds decreased the muscle–tendon unit stiffness of the hamstrings.

The effect of static stretching on the muscle–tendon unit stiffness is affected by the stress of the stretching. The stress of static stretching increases with stretching duration and intensity. Therefore, static stretching of longer duration^5,6,17^ or higher intensity^14–16^ is effective for decreasing the muscle–tendon unit stiffness. However, Fukaya et al. compared the effect of high-intensity with shorter duration (120%ROM for 100 s) and low-intensity with longer duration (50%ROM for 240 s) static stretching on the muscle stiffness of the medial gastrocnemius and showed larger decreases in the muscle stiffness after high-intensity with shorter duration static stretching, even if the same stretching load was applied^18^. Previous studies that examined the effects of high-intensity static stretching decided its intensity based on ROM^14,18,19^ or POD^15,16^. ROM was defined as the maximum angle without pain^14,18,19^. Both ROM and POD are indicators that depend on the subject’s sense and may not be sufficient as objective indicators of stretching intensity. Therefore, it is unclear how much objective stress the muscle received by high-intensity static stretching.

The force applied to the muscle during static stretching can be measured by using passive torque during stretching^20–22^. The passive torque during static stretching gradually decreases over time, which is called stress relaxation^23–25^. Therefore, the stress of static stretching can be quantified by using the static stretching load, which is calculated from the passive torque throughout the stretching. It is possible that the mechanisms in which high-intensity static stretching is effective in decreasing the muscle–tendon unit stiffness of the hamstrings are revealed by analyzing the relationship between the static stretching load and changes in flexibility after high-intensity static stretching.

Therefore, the purpose of the present study was to examine the association between the static stretching load and changes in the muscle–tendon unit stiffness of the hamstrings after two different intensities of static stretching.

## Methods

### Participants

Twelve recreationally active men (21.0 ± 0.8 years, 169.5 ± 8.0 cm, 62.4 ± 6.4 kg) were recruited. Participants who were competitive athletes, who performed regular intensive flexibility or strength training, or who had a history of lower limb pathology were excluded. During the experimental period, all participants were instructed not to perform resistance or flexibility training of the lower limbs. Previous studies that examined the effects of high-intensity static stretching reported large effect sizes for the muscle–tendon unit stiffness of the hamstrings^15,16^. Therefore, the sample size of the muscle–tendon stiffness was calculated with a power of 95%, alpha error of 0.05, and effect size f of 0.40 (large) using G*Power 3.1 software (Heinrich Heine University, Düsseldorf, Germany), and the results showed that the requisite number of participants for this study was 12. All participants were informed of the requirements and risks associated with their involvement in this study and signed a written informed consent document. The study was performed in accordance with the Declaration of Helsinki (1964). The Ethics Committee of Kobe International University approved the study (Procedure #160).

### Procedure

The participants underwent two different intensities of static stretching (100%POD and 120%POD) in the hamstrings, in random order. They visited two times, on separate days, with an interval of one week between visits. To evaluate any alteration of the flexibility of the hamstrings in the dominant leg (ball kicking preference), the knee extension ROM, passive torque at end ROM, and muscle–tendon unit stiffness were measured before and immediately after each bout of static stretching. The static stretching load was calculated from the passive torque throughout static stretching. In addition, to measure stretching pain, numerical rating scale (NRS) was investigated. The experiment was performed in a university laboratory, where the temperature was maintained at 25 degrees C.

### Flexibility assessment

The flexibility assessment was performed in the same fashion as previous studies^14–16^. A previous study reported that the reliability of the measurements used in this study was acceptable^14^. An isokinetic dynamometer machine (CYBEX NORM, Humac, California, USA) was used in the present study. This study used a sitting position in which the hip joint was flexed, which has been shown to efficiently stretch the hamstrings^14^. The participants were seated on a chair with the seat tilted maximally, and a wedge-shaped cushion was inserted between the trunk and the backrest, which set the angle between the seat and the back at approximately 60 degrees. The previous study, which used the same assessment, reported that the average angle of hip flexion was 111.2 ± 2.5 degrees^14^. The chest, pelvis, and right thigh were stabilized with straps. The right knee joint was aligned with the axis of the rotation of the isokinetic dynamometer machine. The lever arm attachment was placed just proximal to the malleolus medialis and stabilized with straps. In the present study, reported knee angles were measured using the isokinetic dynamometer. A 90-degree angle between the lever arm and floor was defined as 0 degrees of knee flexion/extension. The participants were instructed to relax during the flexibility assessment. Participants underwent pre-flexibility measurements after more than 5 min of sitting.

### Knee extension ROM

The knee joint was passively extended from 0 degrees to the maximum angle without pain at 5 degrees/second. ROM was defined as the range from 0 degrees to the maximum knee extension angle.

### Passive torque at end ROM

The passive torque and the knee extension angle during ROM measurements were recorded in the isokinetic dynamometer and were exported a personal computer to analyze the passive torque at end ROM and muscle–tendon unit stiffness. The passive torque at maximum knee extension angle (end ROM) both before and after static stretching was used for further analyses.

### Calculation of muscle–tendon unit stiffness

The muscle–tendon unit stiffness was defined as the value of the slope of the regression line that was calculated from the torque–angle relationship using the least-squares method^14,26^. The muscle–tendon unit stiffness was calculated from the same knee extension angle range before and after stretching. The calculated knee extension angle range was defined as the angle from the 50% maximum knee extension angle to the maximum knee extension angle measured before stretching^5,14,16^. However, if the maximum knee extension angle measured after static stretching was smaller than that before stretching, the muscle–tendon unit stiffness before and after stretching was calculated from the 50% maximum knee extension angle to the maximum knee extension angle measured after stretching^14,16^.

### NRS

The level of pain during static stretching (first and second repetitions) and immediately after the post measurement (post-measurement) was quantified by an 11-point NRS that ranged from 0 (no pain) to 10 (worst imaginable pain)^14,16^.

### Static stretching

Static stretching was performed on the isokinetic dynamometer in the same fashion as the measurement of the knee extension ROM^14–16^. Static stretching for 60 s (30 s of stretching with 30 s intervals) was performed at two different intensities based on the POD of each participant (100%POD and 120%POD intensity). At 100%POD, the angle was set just prior to the POD^14–16^. At 120%POD, the angle was set to 1.2 times the POD. The participants were instructed to relax during each stretch.

### Calculation of static stretching load

**Figure 1:**
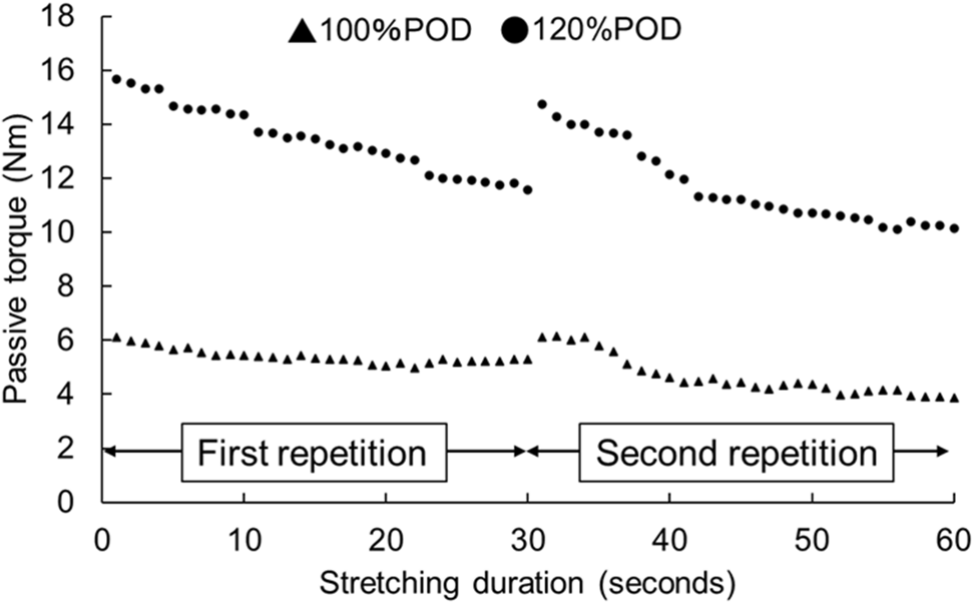
Passive torque during stretching interventions.

The passive torque during static stretching was recorded in the dynamometer machine and was exported to a personal computer to calculate the static stretching load. The static stretching load was the sum of the passive torque during static stretching for 60 s (Fig. 1).

### Statistical analyses

All variables except NRS were described as mean ± standard deviation in the present study. NRS was described as a median (interquartile range). The statistical analyses were performed according to previous studies^15,16^. For the knee extension ROM, passive torque at end ROM, and muscle–tendon unit stiffness, a two-way repeated measures ANOVA was used to examine the effects of intervention (100%POD versus120% POD) and time (pre versus post). For NRS, a two-way repeated-measures ANOVA was used to examine the effects of intervention (100%POD versus120% POD) and time (first repetition versus second repetition versus post-measurement). If a significance was detected, post hoc analyses using Bonferroni’s test were performed. A paired t-test was used to examine the difference in the static stretching load between 100%POD and 120%POD. Pearson’s correlation coefficient was conducted between the static stretching load and relative changes of the knee extension ROM, passive torque at end ROM, and muscle–tendon unit stiffness. The analyses were performed using SPSS version 25 (SPSS, Inc., Chicago, IL, USA). Differences were considered statistically significant at an alpha level of *p* < 0.05.

## Results

### Knee extension ROM

**Figure 2:**
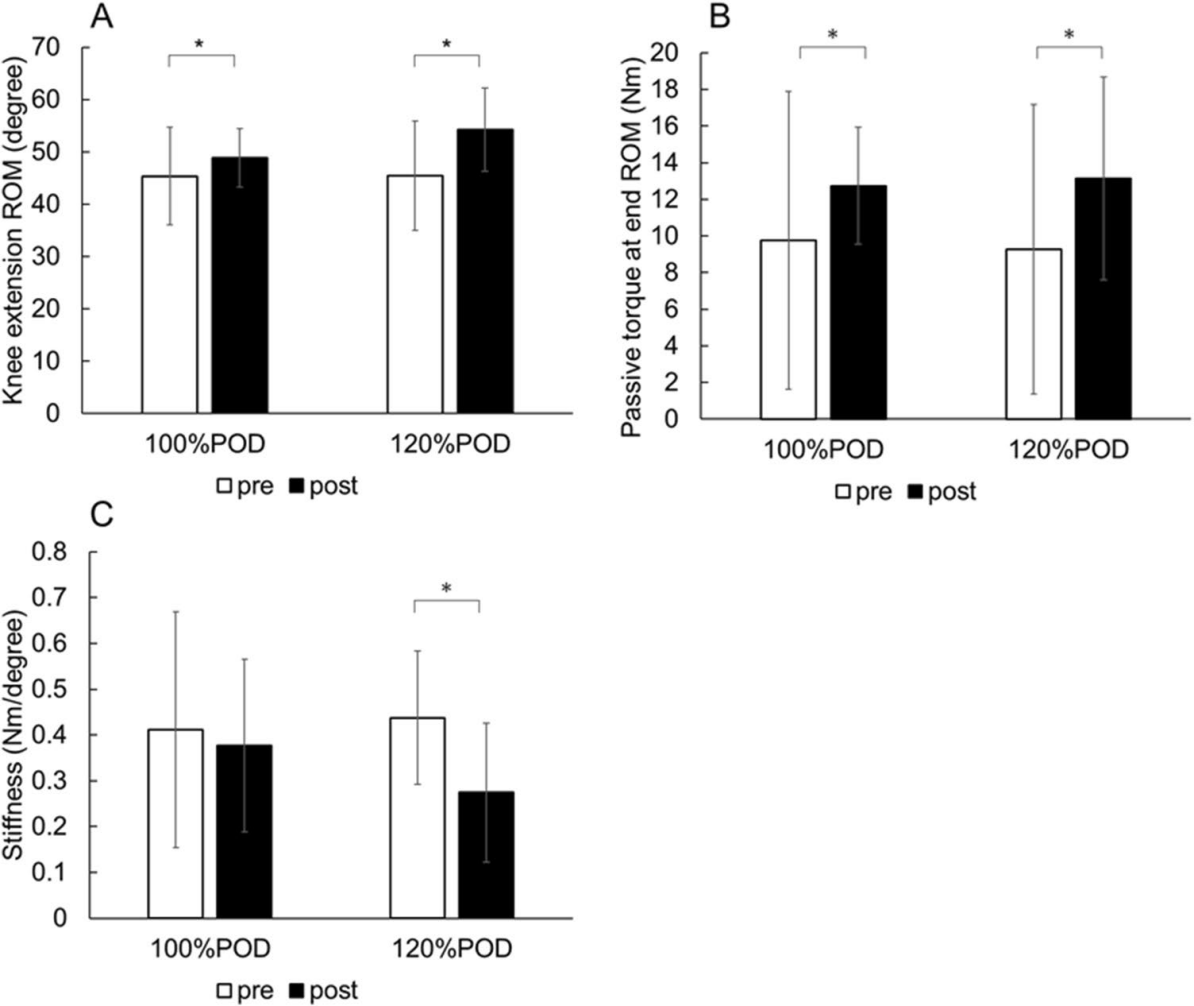
Changes in knee extension ROM (**A**), passive torque at end ROM (**B**), and muscle–tendon unit stiffness (**C**). Data were represented as mean ± standard deviation. * *p* < 0.01 (pre versus post). ROM: range of motion.

There was a significant two-way interaction (intervention × time, *p* < 0.01, partial eta squared = 0.46) (Fig. 2). The knee extension ROM increased in both intensities (both *p* < 0.01).

### Passive torque at end ROM

There was no significant two-way interaction (intervention × time, *p* = 0.58, partial eta squared = 0.02) and no main effect for intervention (*p* = 0.99, partial eta squared < 0.01), but there was a significant main effect for time (*p* < 0.01, partial eta squared = 0.49) (Fig. 2). The passive torque at end ROM increased in both intensities (*p* < 0.01).

### Muscle–tendon unit stiffness

There was a significant two-way interaction (intervention × time, *p* < 0.01, partial eta squared = 0.37) (Fig. 2). The muscle–tendon unit stiffness decreased in the 120%POD (*p* < 0.01), but it did not change in the 100%POD (*p* = 0.42).

### Static stretching load

The static stretching loads of 100%POD and 120%POD were 234.3 ± 192.1 Nm and 469.3 ± 149.5 Nm, respectively. The static stretching load of the 120%POD was significantly higher than that of 100%POD (*p* < 0.01).

### Correlations between static stretching load and measurements of flexibility

**Figure 3:**
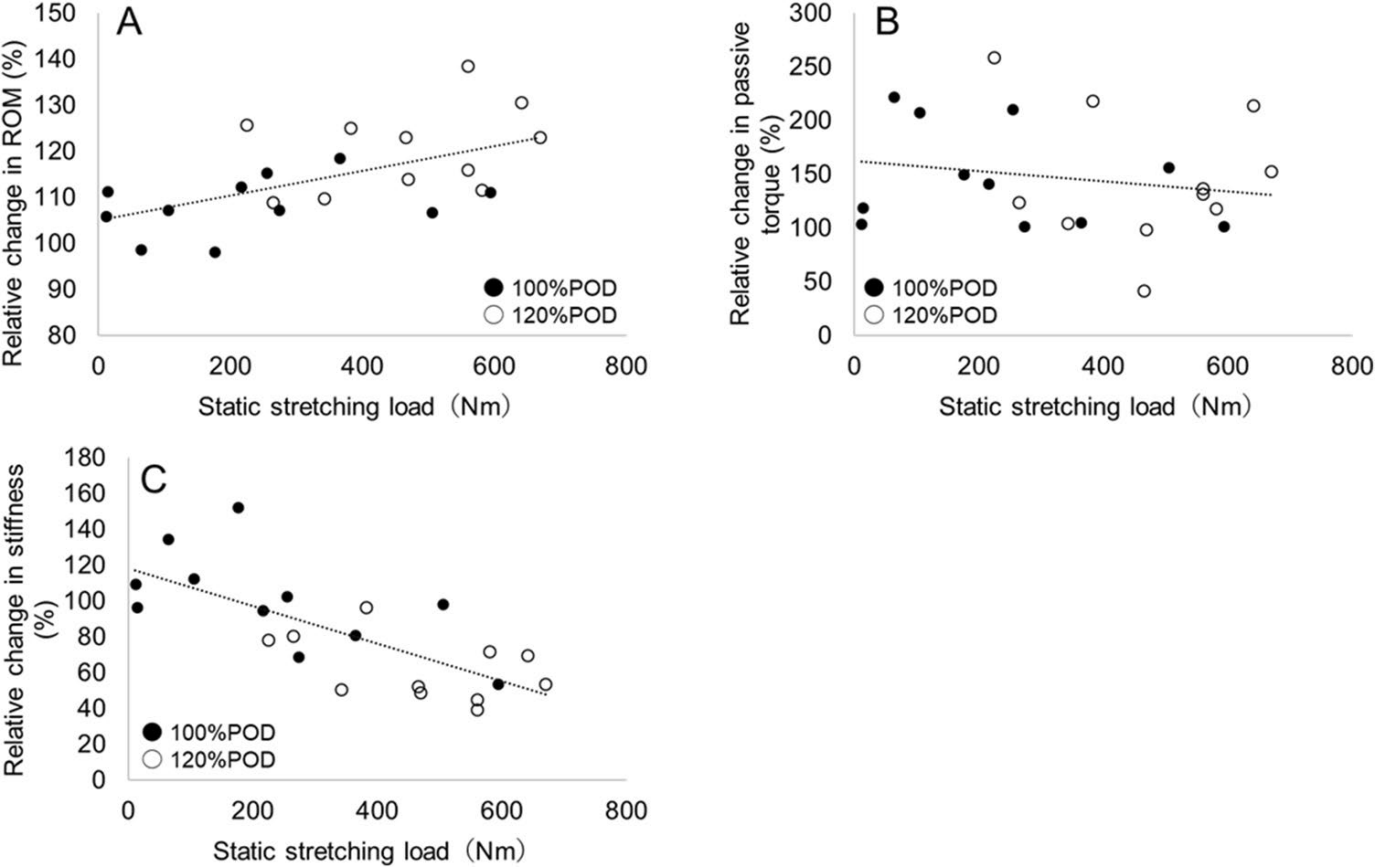
Correlation between static stretching load and relative changes in knee extension ROM (**A**), passive torque at end ROM (**B**), and muscle–tendon unit stiffness (**C**). ROM: range of motion.

There were significant correlations between the static stretching load and relative changes in the knee extension ROM (r = 0.56, *p* < 0.01) and muscle–tendon unit stiffness (r = -0.76, *p* < 0.01) (Fig. 3). However, there was no significant correlation between the static stretching load and relative change in the passive torque at end ROM (r = -0.18, *p* = 0.42).

### NRS

There was a significant two-way interaction (intervention × time, *p* < 0.01, partial eta squared = 0.64) (Table 1). In first and second repetitions, NRS of the 120%POD indicated higher values than those of 100%POD (both *p* < 0.01). In the 120%POD, NRS in the first and second repetitions indicated higher values than that post-measurement (both *p* < 0.01).

**Table 1:** Time course of change in NRS.

	First repetition	Second repetition	Post-measurement
100%POD	1 (0—1)	1 (0—1)	0 (0—0)
120%POD	5 (4 -5) *^,†,‡^	4 (3—4)*^,‡^	0 (0—0)

Data were described as median (25%—75%). * *p* < 0.01 versus value at 120%POD in the post-measurement. ^†^ *p* < 0.05 versus value at 120%POD in the second repetition. ^‡^* p* < 0.01 versus value at 100%POD at the same time.

## Discussion

The present study investigated the association between static stretching load and the flexibility of the hamstrings after static stretching. The results of the present study showed that the static stretching load in the 120%POD was higher than that of 100%POD (approximately two times higher). Moreover, there were significant correlations between the static stretching load and the relative changes in the knee extension ROM and muscle–tendon unit stiffness of the hamstrings. These data indicated that static stretching load was important for decreasing the muscle–tendon unit stiffness of the hamstrings, and high-intensity static stretching is useful for decreasing the stiffness because of its high static stretching load. This is the first article that has examined the effects of static stretching load on the flexibility of the hamstrings.

In the present study, the knee extension ROM increased in both intensities. Changes in ROM after static stretching are attributed to the changes in stretching tolerance^20,22,27,28^ and the muscle–tendon unit stiffness^5,6,29,30^. In the present study, the passive torque at end ROM was measured in order to examine the change in stretching tolerance^20,22,27,28^. The results of the present study showed that the passive torque at end ROM significantly increased in both intensities, although the muscle–tendon unit stiffness significantly decreased only in the 120%POD. More than 180 s of static stretching at the intensity of 100%POD is needed to decrease the muscle–tendon unit stiffness of the hamstrings^5,6^. On the other hand, static stretching at the intensity of 120%POD or more decreases the muscle–tendon unit stiffness even if the duration of the stretching is 20 or less seconds^15,16^. Therefore, the results of the present study indicated that 60 s of static stretching at the intensity of 100%POD increased the knee extension ROM due to increasing stretching tolerance, not a change in the muscle–tendon unit stiffness. On the other hand, 60 s of static stretching at the intensity of 120%POD increased the knee extension ROM due to both increasing stretching tolerance and decreasing the muscle–tendon unit stiffness of the hamstrings.

In the present study, the static stretching load of the 120%POD was approximately two times higher than that of the 100%POD, even though the knee extension angle during the stretching was set at 1.2 times higher. It was supposed that the difference between the static stretching load and knee extension angle during stretching was related to the torque–angle curve during passive joint movement. In passive joint movement, the passive torque does not increase in the approximately first 50% of the ROM, but it increases thereafter^21,31^. Therefore, in the 120%POD, the knee extension angle during the stretching was set to 1.2 times the 100%POD, but the hamstrings received a greater static stretching load than the angle change. This difference between stretching angle and static stretching load may be related to the discrepancy in the results in previous studies^14,19^. It was reported that 3 min of static stretching at the intensity of 120%ROM significantly decreased the muscle–tendon unit stiffness of the hamstrings^14^, but it did not change the muscle stiffness of the rectus femoris because it was overly stressful^19^. In the hamstrings, the knee extension angle of 120%ROM is approximately 10 degrees greater than that of 100%ROM^14^, while in the rectus femoris the knee flexion angle of 120%ROM is approximately 20 degrees greater than that of 100%ROM^19^. Therefore, although the stretching intensity was set at the same intensity of 120%ROM in both previous studies^14,19^, the static stretching load received by the hamstrings and rectus femoris may be quite different. Caution may be necessary when athletes decide the intensity of static stretching because the targeted muscle receives greater stress than the stretching angle.

In the present study there were significant correlations between the static stretching load and relative changes in the knee extension ROM and muscle–tendon stiffness of the hamstrings. Furthermore, all participants with high static stretching load decreased the muscle–tendon unit stiffness regardless of stretching intensity. On the other hand, there was no significant correlation between the static stretching load and relative change in the passive torque at end ROM. The static stretching load was affected by both stretching duration and intensity, although the present study compared two different intensities of static stretching. In previous studies, it was reported that changes in ROM^15,16,32,33^ and the muscle–tendon unit stiffness^5,6,15,16^ of the hamstrings increase with stretching duration and intensity. On the other hand, studies that examined the change in the passive torque at end ROM of the hamstrings after high-intensity static stretching have shown inconsistent results^14–16^. The results of the present study indicated that the static stretching load had significant effects on the changes in the muscle–tendon unit stiffness of the hamstrings regardless of stretching intensity, and high-intensity static stretching is useful for decreasing the stiffness because of its high static stretching load.

In the present study, the median values of NRS during static stretching were 1 and 4–5 in the 100%POD and 120%POD, respectively, although it was 0 post-measurement in both intensities, which is consistent with the previous studies^15,16^. Previous studies reported that the pain during high-intensity static stretching was moderate to severe, but it disappeared after stretching^15,16^. These data indicated that the high-intensity static stretching used in the present study was as safe as high-intensity static stretching used in the previous studies^15,16^.

There were some limitations in the present study. Firstly, the present study used the two different intensities of static stretching in order to investigate the association between the static stretching load between the flexibility of the hamstrings. Therefore, the effects of stretching duration on the static stretching load is unclear. Secondly, the present study measured the muscle–tendon unit stiffness of the hamstrings. However, it was suggested that the effects of high-intensity static stretching on the muscle stiffness may differ depending on its targeted muscle^19^. Therefore, the effects of the static stretching load need to be examined in muscles other than the hamstrings.

In conclusion, the static stretching load of 120%POD was significantly higher than that of 100%POD. There were significant correlations between the static stretching load and the relative changes in the knee extension ROM and muscle–tendon unit stiffness of the hamstrings. However, there was no significant correlation between the static stretching load and the relative change in the passive torque at end ROM. These data suggested that the static stretching load had significant effects on the increase in the knee extension ROM and decrease in the muscle–tendon unit stiffness of the hamstrings, and high-intensity static stretching was useful for changes in the flexibility of the hamstrings because of its high static stretching load.

## Data Availability

The datasets generated during and/or analyzed during the current study are available from the corresponding author on reasonable request.
